# MitoCommun: a database for decoding mitochondrial communication networks

**DOI:** 10.1186/s12864-026-12549-6

**Published:** 2026-01-16

**Authors:** Xueyan Wu, Danlei Chen, Jitong Feng, Xueying Bu, Shengbo Wu, Jianjun Qiao

**Affiliations:** 1 School of Synthetic Biology and Biomanufacturing, State Key Laboratory of Synthetic Biology, Key Laboratory of Systems Bioengineering (Ministry of Education), Tianjin University, Tianjin, 300350 China; 2 Zhejiang Institute of Tianjin University (Shaoxing), Shaoxing, 312300 China

**Keywords:** Mitochondria, Signaling networks, Organelle communication, Intercellular crosstalk, Signaling transduction, Mitochondrial-nuclear interaction

## Abstract

Mitochondria are highly dynamic signaling organelles that engage in continuous bidirectional communication with the cytoplasm and participate in biological processes such as cell death, inflammation, and epigenetic modification. Mitochondria receive, process and export various types of signals, ranging from metabolites to non-coding RNAs. Despite their central importance, progress in understanding mitochondrial signaling has been hampered by the fragmented characterization of their communication networks. In this study, we present MitoCommun, a manually curated database comprising 580 mitochondria-derived signaling events from five model species: *Homo sapiens*, *Mus musculus*, *Rattus norvegicus, Saccharomyces cerevisiae*, and *Caenorhabditis elegans*. Specifically, MitoCommun features 233 well-annotated mitochondrial signaling molecules, defined by either mitochondrial origin or specific organelle localization. These molecular entities have been systematically paired with their receptors and functionally mapped into explicit pathways and interaction networks. Furthermore, we have reconstructed signaling networks across phylogenetically divergent species, functionally distinct biological processes, and inter-organelle communication systems, yielding comprehensive topological maps of mitochondrial signaling cascades. By integrating these resources, MitoCommun establishes a foundational framework for decoding mitochondrial communication and serves as a versatile, user-friendly platform for exploring organelle signaling networks, which is freely accessible at http://mitocommun.qscn.online/.

**Supplementary Information:**

The online version contains supplementary material available at 10.1186/s12864-026-12549-6.

## Highlights

● Comprehensive data were curated for different mitochondrial communications.

● A user-friendly database was developed for browsing and searching the results.

● Multiple signals and pathways were collected, combining detailed cell stresses.

● Mitochondrial signaling networks were integrated across organisms, organelles and functions.

**Supplementary Information:**

The online version contains supplementary material available at 10.1186/s12864-026-12549-6.

## Introduction

Mitochondria are dynamic, eukaryotic-specific organelles that harbor a diverse repertoire of nuclear-encoded proteins, extending their functional repertoire far beyond their canonical role in cellular energetics [1–3]. Beyond regulating cellular proliferation, differentiation, and metabolic activities, mitochondria also govern epigenetic modifications [4], participate in inflammatory responses [5, 6], drive immune activation [7], and critically determine cell survival and fate [8]. They play pivotal roles in endocrine signaling, oxidative stress regulation, cellular physiology, homeostasis maintenance [8, 9], osmotic balance, and tumor metastasis, while also serving as central arbiters of cellular senescence and programmed cell death [3, 10, 11]. Generally, mitochondria maintain functional homeostasis through autonomous regulation of core processes including energy metabolism, dynamic fusion/fission cycles, mitophagy, oxidative stress responses, calcium signaling [12], and reactive oxygen species (ROS) balance [13]. Furthermore, they participate in sophisticated communication networks, engaging in bidirectional inter-organellar signaling (particularly with the nucleus), intercellular communication, and systemic signal transduction pathways, underscoring their integrative role in cellular and organismal physiology [14, 15].

Mitochondria function as stress-sensing organelles that maintain their activity and homeostasis through autonomous regulatory mechanisms [16, 17]. Their dynamic fusion-fission cycles generate metabolically specialized subpopulations that spatially organize oxidative phosphorylation and reductive biosynthesis, enabling adaptive responses to fluctuating nutritional states and energy demands [18]. Mitochondrial quality control systems, including fusion, fission, and mitophagy, precisely regulate organellar quantity and integrity by eliminating damaged ones [19]. When damage exceeds repair capacities, mitochondria initiate stress responses such as the mitochondrial unfolded protein response (UPRmt), generating cascades of stress signals that recalibrate cellular homeostasis [20]. Under irreparable stress conditions, compromised membrane integrity triggers the release of mitochondrial damage-associated molecular patterns (mito-DAMPs), which subsequently orchestrate cell death, inflammatory cascades, and immune activation [6, 7, 11, 21–23]. Through these layered regulatory mechanisms, mitochondria not only detect stress but also integrate and propagate systemic signals [24, 25], effectively functioning as endocrine-like signaling hubs that coordinate cellular and organismal physiology [26, 27].

Mitochondria serve as integrative signaling hubs capable of receiving, processing, and transducing diverse molecular signals, including metabolites, proteins, and nucleic acids, thereby coordinating cellular responses [14, 28]. Their extracellular communication occurs through multiple modalities, such as mitochondrial-derived peptides (MDPs), mitochondrial-associated proteins, metabolic intermediates, mitochondrial DNA (mtDNA), membrane components, cytokines, steroids and mitochondria-derived vesicles (MDVs) [29, 30]. At the inter-organelle level, mitochondria engage in sophisticated cross-talk with other cellular compartments through specialized mechanisms [31–33]. During endoplasmic reticulum (ER) stress, mitochondria coordinate cellular adaptation through a tripartite mechanism involving bioenergetic provisioning, stress-signaling pathway activation, and dynamic architectural remodeling [34]. Particularly noteworthy is the bidirectional mitochondrial-nuclear communication axis [35–37], where mitochondrially-derived signaling molecules interact with nuclear transcription factors to orchestrate genome-wide expression networks [38, 39]. This evolutionarily conserved dialogue critically regulates cellular homeostasis, environmental adaptation, and fate determination [40].

Given mitochondria's pivotal role in orchestrating diverse cellular phenotypes, some efforts have been made to curate mitochondrial data, yielding several specialized resources. InterMitoBase was an annotated database of protein–protein interactions for human mitochondria [41]. MitoMap serves as the reference database for human mitochondrial genome annotation [42], while MitoCarta3.0 provides comprehensive coverage of human mitochondrial proteins, including submitochondrial localization and pathway annotations [43]. Metabolic databases such as Human Metabolome Database (HMDB) and Virtual Metabolic Human (VMH) offer organelle-specific metabolite distributions and metabolic network mapping, respectively [44, 45], with the Yeast Metabolome Database (YMDB) enabling systematic identification of yeast metabolites and their signaling roles [46]. The KEGG database further facilitates comprehensive retrieval and annotation of mitochondrial pathways across species [47].

Despite the aforementioned valuable resources, a critical gap persists in the systematic integration of mitochondrial stress responses and communication networks. Current knowledge remains fragmented, with existing compilations often limited to schematic representations of select pathways rather than framework-based organization. Notably absent is a unified platform that integrates cross-species signaling molecules, pathways, and regulatory functions into coherent networks. This deficiency becomes increasingly pressing as mitochondrial research accelerates, with proteomic and metabolomic datasets expanding rapidly. The field urgently requires a dedicated mitochondrial signaling resource database to facilitate efficient mining and validation of different mitochondrial regulatory mechanisms.

In this study, to systematically decode mitochondrial communication networks, we present an integration combining computational curation, network visualization, and functional analysis of mitochondrial signaling mechanisms across evolutionarily distant model systems. Specifically, we focused on three taxonomic groups: the budding yeast *Saccharomyces cerevisiae* (*S. cerevisiae*), the nematode *Caenorhabditis elegans* (*C. elegans*), and three mammalian species—*Homo sapiens, Mus musculus* and *Rattus norvegicus*. Humans constitute the primary source of mitochondrial signaling literature, while mouse and rat are the most widely used in vivo models in which many human mitochondrial pathways have been mechanistically validated. Given the extensive conservation of core mitochondrial processes across these mammals, we aggregated these three species into a single "mammalian" group for comparative analyses, while recognizing that species-specific physiological and metabolic differences are likely to exist. Through rigorous curation and manual verification, we established a high-confidence database comprising 580 non-redundant mitochondrial signaling communication entries, complemented by an interactive web platform featuring advanced query and visualization capabilities (http://mitocommun.qscn.online/). We first reconstructed species-specific signaling pathway maps and then compared them across taxa to identify evolutionarily conserved versus lineage-specific modules. Building upon this foundation, we performed a systematic visualization of mitochondrial communication networks across different species, biological functions, and inter-organelle interactions, revealing both deeply conserved principles and clade-specific regulatory architectures. Finally, this comparative analysis not only yields organism- and process-specific mitochondrial communication networks but also provides mechanistic insights with potential applications in fungal biology and mammalian systems, particularly for understanding conserved signaling principles and developing targeted interventions.

## Materials and methods

### Raw data collection

We systematically retrieved mitochondrial communication-related information including signaling pathways, biological processes, experimental results, tables, and figures from peer-reviewed publications covering *S. cerevisiae*, *C. elegans*, and three mammalian species—*Homo sapiens, Mus musculus* and *Rattus norvegicus*. Literature searches were performed primarily in PubMed and Web of Science using combinations of the terms "mitochondria/mitochondrial", "signal/signaling", "communication", "mitokines", "retrograde signaling", "UPRmt", "cell death", "inflammation", "immunity", "crosstalk", "nucleus", "stress", and "mitochondrial dysfunction", etc. To minimize bias introduced by keyword selection and to capture historically important pathways, we also used high-impact review articles on mitochondrial signaling published in the past decade as entry points and performed backward citation tracking to identify earlier primary studies that might not explicitly contain the chosen keywords in their titles or abstracts. During literature collection, we categorized signaling molecules into nucleic acids, polypeptides/proteins, and small-molecule compounds, with further subclassification and refinement based on existing studies [48–50]. For each relevant publication, information on organism, signaling types, signal sources, receptors, experimental context, and functional phenotypes was manually extracted from both textual descriptions and graphical content (e.g. pathway diagrams and summary schematics). Given the breadth and rapid growth of the field, it is unlikely that all existing peer-reviewed articles were captured. Our dataset therefore represents a carefully curated yet non-exhaustive compendium of mitochondrial signaling events up to July 2025, with a particular emphasis on experimentally validated pathways and evolutionarily conserved mechanisms. The complete list of primary publications curated into MitoCommun is provided as Additional file 1 (Excel spreadsheet), which includes authors, publication year, PubMed ID, DOI, and the type of data curated for each study.

### Reference database integration

To comprehensively augment mitochondrial communication pathway information, we systematically integrated multi-omics data from established biological databases (Table 1). Specifically, protein datasets were curated from UniProt [51] through targeted extraction of mitochondrial protein entries, with additional mitochondrial-specific resources incorporated from MitoCarta and KEGG pathway databases. The metabolite-relevant data integration involved retrieval of mitochondrial metabolites and associated metabolic pathways, which were mainly collected from the Virtual Metabolic Human (VMH) database and the Human Metabolome Database (HMDB). Specialized mitochondrial-derived molecules, including non-peptide metabolites such as steroid hormones, were acquired from the hormone-specific repository HmrBase2 [52]. For protein signaling network reconstruction, we employed rigorous interaction mapping using both STRING [53] and BioGrid [54] databases to identify and validate receptor-protein interactions. Small molecule signaling pathways were elucidated through comprehensive matching of metabolites with their cognate receptors using BindingDB [55], enabling systematic reconstruction of metabolite-receptor interaction networks. This multi-layered data integration strategy ensures robust coverage of mitochondrial communication components across molecular scales, from proteins and metabolites to their interacting partners and pathway contexts.

**Table 1:** Database resource

Database	Description	URL	Refer
STRING	Protein–Protein Interaction Networks	http://string-db.org/	[53]
BioGrid	Protein, Genetic and Chemical Interactions	http://thebiogrid.org/	[54]
BindingDB	Protein–ligand binding data	https://www.bindingdb.org/bind/index.jsp	[55]
VMH	Information on human and gut microbial metabolism	https://www.vmh.life/	[45]
HMDB	Small molecule metabolites in the human body	https://hmdb.ca/	[44]
YMDB	Small molecule metabolites found in or produced by *S. cerevisiae*	https://www.ymdb.ca/	[46]
Hmrbase2	Peptide and non-peptide hormones and their receptors	https://webs.iiitd.edu.in/raghava/hmrbase2/	[52]
KEGG	Integrates genomic, chemical, and functional information of biological systems	https://www.genome.jp/kegg/	[47]
MitoCarta3.0	Genes encoding mitochondrial proteins, with sub-mitochondrial localization and pathway annotations	http://www.broadinstitute.org/mitocarta	[43]
UniProt	Protein sequence and functional information	https://www.uniprot.org/	[51]

### Data processing, filtering and statistical analysis

**Fig. 1:**
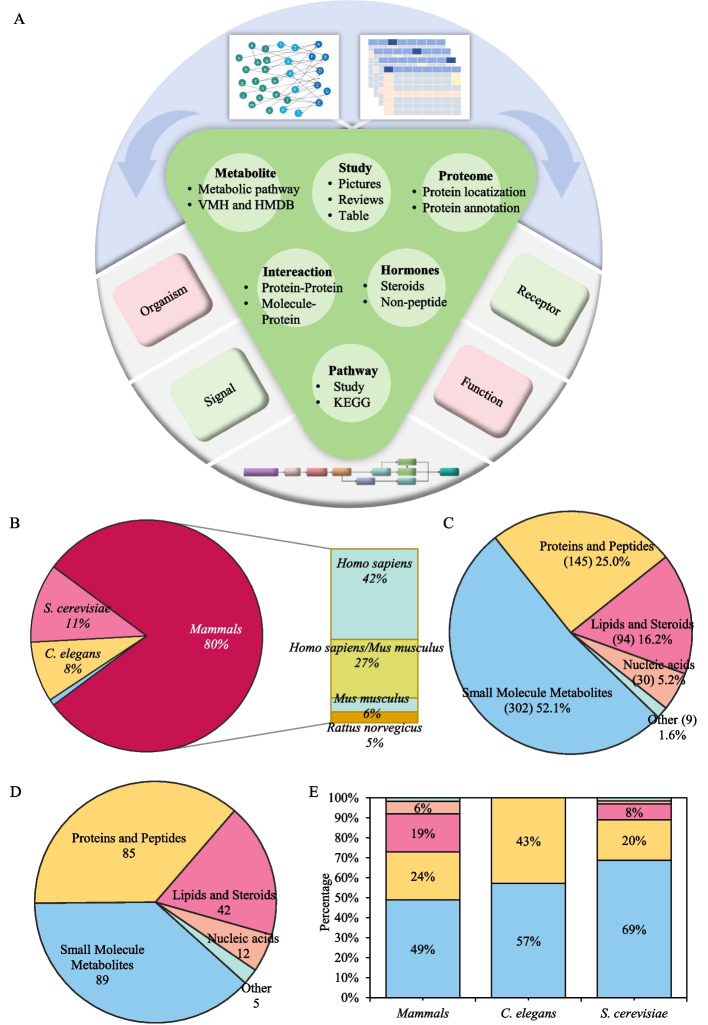
Data processing and database establishment. **A**. Data were collected from literature and multiple databases (including metabolite- and protein-related databases), with entries containing species, signaling molecules, receptors, and functional annotations. Statistical analysis of signaling entries is shown. **B**. Species distribution of database entries. **C**. Percentage and quantity of entries corresponding to five signal types. **D**. The quantity of each signal type in all signals. **E**. Distribution comparison of five types of signal corresponding entries among mammals, *S. cerevisiae*, and *C. elegans*

All data extraction, filtering and integration steps were performed manually. For literature-derived entries, information was first recorded in spreadsheet tables (Microsoft Excel). Basic functions (e.g. conditional filtering, text search and lookup operations) were then used to remove duplicates, harmonize gene/protein and metabolite names and match small molecules to their cognate receptors using unique identifiers from external databases (e.g. HMDB IDs, UniProt accessions, KEGG compound IDs). For each candidate signaling event, we required at least one peer-reviewed study providing experimental support for the reported molecule-receptor-function relationship in the specified organism. Entries that were ambiguous, poorly supported or based solely on in silico predictions without experimental validation were excluded at this stage. To reduce redundancy, multiple studies reporting the same signaling interaction under similar conditions were merged into a single curated entry with cross-references to all supporting publications. Summary statistics for Fig. 1 were derived directly from the curated tables in Excel by counting non-redundant signaling molecules and pathways for each species (or taxonomic group) and each signal type.

### Database implementation

The MitoCommun platform was implemented using a standard LAMP-based architecture complemented by a lightweight Python backend. The curated and integrated data were stored in a MySQL 8.0.38 database (InnoDB storage engine and UTF-8 encoding). The web front end was built with HTML5, CSS3 and JavaScript (ES2023), while server-side logic and application programming interfaces (APIs) were implemented in PHP 8.3.10 and Python 3.10 using the Flask 3.2.1 framework together with pandas 2.2.3 for data handling. Interactive visualization was implemented with ECharts 5.5.0, and user-interface components were developed using jQuery 3.7.1 and Bootstrap 5.3.3. Dynamic result tables with client-side sorting, filtering and export (CSV) were implemented using the DataTables 1.13.8 plug-in. The website is hosted on an Apache 2.4.62 server running a Linux-based operating system. To ensure cross-platform compatibility, the MitoCommun website was tested on the latest versions of Mozilla Firefox, Google Chrome, Microsoft Edge and legacy Internet Explorer modes.

## Results

### Data collection and statistics

Through meticulous manual curation and verification, we systematically extracted mitochondrial-related information from extensive literature sources, experimental results, tables, and figures, encompassing organisms, signaling types, receptors, and corresponding functional phenotypes as illustrated in Fig. 1a. We integrated multiple data sources (Table 1) including VMH, HMDB, KEGG, and STRING. Our comprehensive literature review and database mining yielded 580 mitochondrial signaling entries with associated species, receptors, and functional annotations. This collection includes 233 mitochondrially-derived or localized signaling molecules, featuring canonical representatives such as ROS, ATP, succinate, mtDNA, and HSP60. All 580 entries were subsequently subjected to systematic analysis of their intrinsic attributes and interrelationships. In the following sections, the mechanistic descriptions of individual mitochondrial signaling pathways primarily summarize published experimental findings that were curated into MitoCommun, whereas the cross-species, cross-functional and cross-organelle comparisons (e.g. Figures 3, 4 and 5) represent new integrative analyses based on our systematic reorganization of these data.

As illustrated in Fig. 1b, the dataset exhibits a pronounced species bias, with the aggregated "mammals" group dominating the information entries (approximately 80%, *n* = 462). This group comprises *Homo sapiens, Mus musculus* and *Rattus norvegicus*, with human-derived data constituting the majority of entries and mouse and rat contributing primarily in vivo experimental validation of conserved pathways. Because these three species share highly conserved mitochondrial pathways and signaling components, we aggregated them for the initial comparative analyses presented in Fig. 1, while providing species-resolved information for all entries in the online database. The remaining entries were distributed across the two non-mammalian model organisms, with *S. cerevisiae* (11%, *n* = 64) and *C. elegans* (8%, *n* = 49) collectively contributing 113 entries. While this grouping highlights broad differences between unicellular yeast, invertebrate nematodes and mammals, more fine-grained species-level comparisons will be necessary to fully resolve mammal-specific physiological and metabolic adaptations.

Our final compilation encompassed 233 distinct mitochondrial signaling molecules involved in intercellular communication. These signaling mechanisms were systematically classified into five major categories: Small Molecule Metabolites, Proteins and Peptides, Lipids and Steroids, Nucleic Acids, and Other. As illustrated in Fig. 1c and d, metabolic signals predominated, comprising 89 small molecule metabolites corresponding to 302 data entries (52.1% of total database entries). Protein and peptide signals accounted for 25% (85 distinct molecules and 145 data entries), followed by lipids and steroids at 16.2% (42 molecules and 94 data entries). Nucleic acid signals represented 5.2% of total entries (12 molecules and 30 data entries), with the remainder being physicochemical signals types. Species-specific analysis (Fig. 1e) demonstrates that metabolic signals constitute the predominant category (> 50% of entries in all species examined). The database reveals striking interspecies divergence in signaling capacity, with *S. cerevisiae* completely lacking hormonal signals, *C. elegans* showing no detectable nucleic acid or lipid and steroid components, and the "mammals" group exhibiting the full spectrum of signal types. Notably, there is a predominance of protein/peptide signals (24%) and lipid and steroid signals (19%) in the "mammals" group, which reflects their expanded receptor repertoire and complex endocrine regulation networks.

This distribution pattern underscores mitochondria's dual role as both metabolic hubs and signaling centers, where metabolic intermediates frequently serve dual functions as signaling molecules. Our integrative analysis reveals mitochondria to be remarkably sensitive signaling organelles capable of diverse molecular communication. Significantly, mitochondria participate actively in steroid hormone biosynthesis, hosting rate-limiting steps in several endocrine pathways. Beyond metabolites, mitochondrial-derived proteins/peptides and nucleic acids can also function as context-dependent signaling molecules, capable of regulating mitochondrial homeostasis and cellular physiology through complex activation and feedback loops.

### Constructing the MitoCommun database

**Fig. 2:**
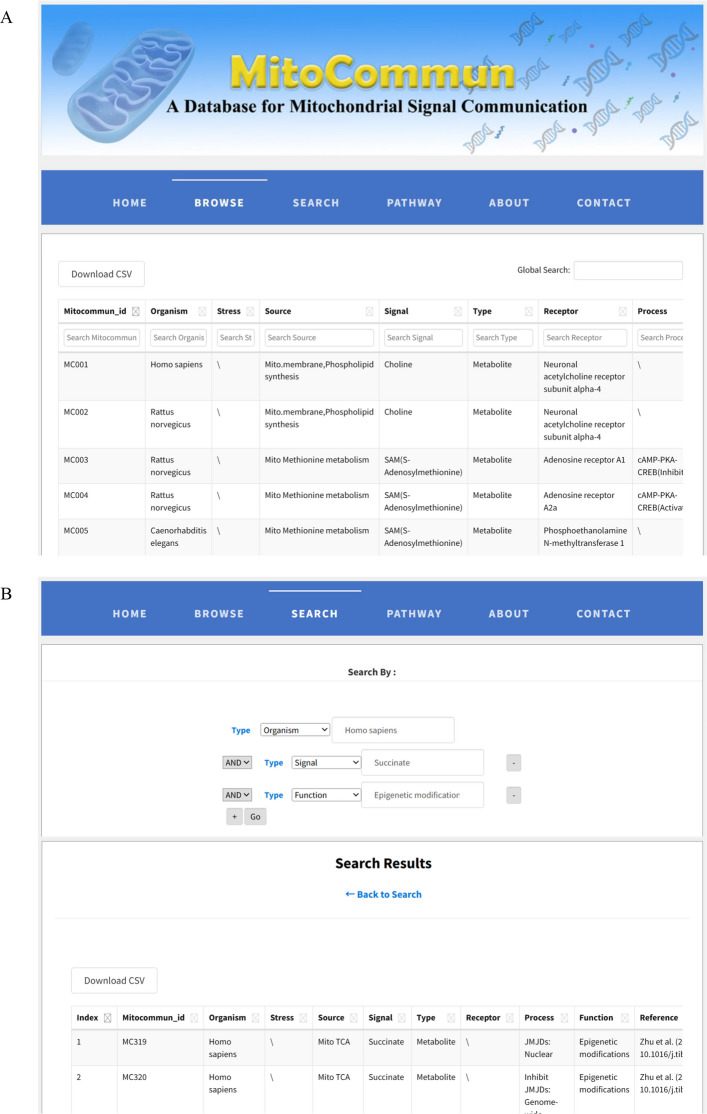
Web interface of the MitoCommun database. **A**. An example Browse view with a general introduction, an interactive table (sortable, filterable) and download options. **B**. An example Search view illustrating the results table and download options after a combined query using criteria such as “organism” and “signal”

Mitochondria can release various signaling molecules, such as ROS, mitochondrial DNA, mitochondrial transcription factors, and metabolites, which can be transmitted into the cytoplasm and the nucleus to influence diverse processes such as gene expression, the cell cycle, and apoptosis. Here, we propose the concept and framework of Mitochondrial Signal Communication (MSC) to systematically decipher and understand mitochondria-derived signaling. On this basis, we constructed the MitoCommun database (Fig. 2), which currently includes 580 curated mitochondrial signaling entries from *Homo sapiens, Mus musculus, Rattus norvegicus*, *S. cerevisiae* and *C. elegans*, and provides user-friendly browsing and searching functions to support various mitochondria-based applications.

The Browse option provides the full collection of curated entries in an interactive table. Each row corresponds to one signaling event and includes the following fields: organism (exact species), stresses, signal source, signal molecules, signal types, receptors, process (signaling pathway), function (regulation, etc.) and reference. The Reference column lists the first author and publication year, along with the digital object identifier (DOI) and the clickable URL (PubMed). Users can browse all the data entries, sort the table by any column, apply text filters that support multi-column combination filtering, and download the results view as CSV files.

In the Search option, an advanced query interface is provided where users can dynamically add multiple query fields, each with a dedicated dropdown menu for criteria such as "Organisms", "Signals", "Receptors", or "Functions". These conditions can be freely combined to construct composite queries, enabling precise retrieval of MSC entries that match their specific research interests. All search boxes are case-insensitive. An auto-complete and fuzzy/partial matching function suggests valid terms from the underlying database. For example, typing "Homo" proposes "*Homo sapiens*", and typing "mus" proposes "*Mus musculus*". To avoid ambiguity, the Organism column in both modules always reports the exact experimental species; generic labels such as "mammals" are not used in the database itself.

The construction of the MitoCommun database provides the basis for the comparative analyses of mitochondrial communication networks across organisms, functions and organelles described in the following sections.

### Mitochondrial signaling pathways in different organisms

**Fig. 3:**
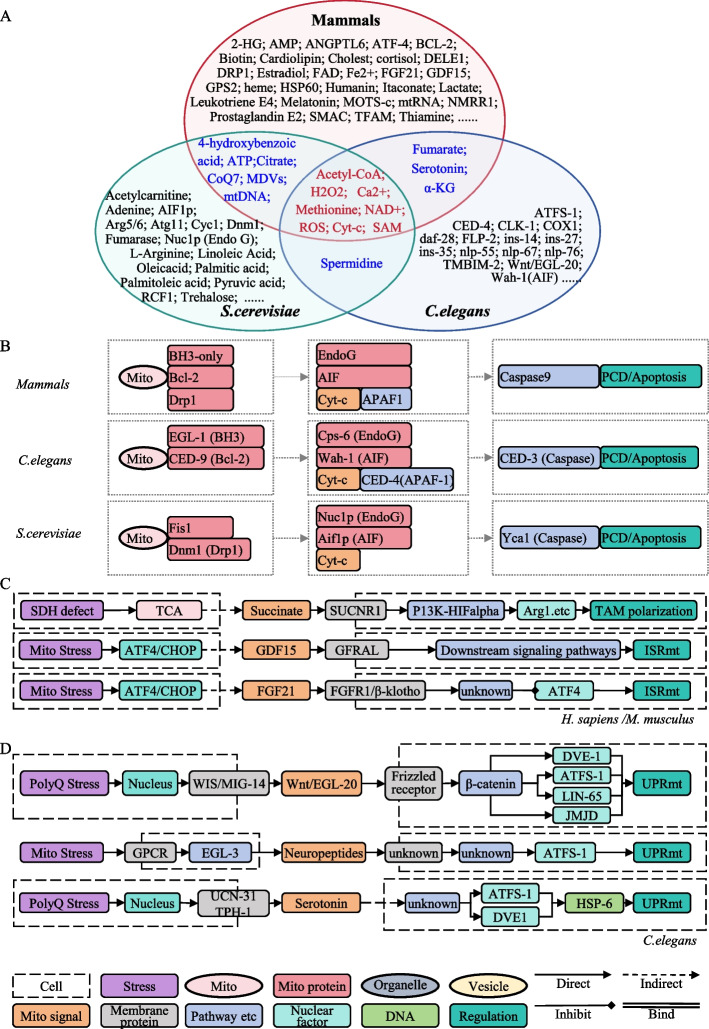
Comparative analysis of mitochondrial signaling pathways across species. **A**. Overview of conserved and species-specific mitochondrial signaling in mammals, *S. cerevisiae*, and *C. elegans*. **B**. Cross-species comparison of apoptotic pathways. **C**. Mammalian-specific inter-tissue mitochondrial communication networks. **D**. Systemic mitochondrial signaling pathways in *C. elegans*. Legend: different shapes and colors distinguish signaling pathway modules. Solid triangle arrows indicate activation or forward signaling; Solid rhombus arrows indicate inhibition; Dashed arrows denote indirect interactions; Double solid lines indicate molecular binding or complex formation. Note: Figs. 4 and 5 share the legend with Fig. 3

Mitochondria originated from an ancestral α-proteobacterial endosymbiont and have since diversified together with their eukaryotic hosts. Comparing mitochondrial signaling networks across evolutionarily distant species is therefore crucial for distinguishing deeply conserved communication principles from lineage-specific adaptations. In MitoCommun, we systematically compiled mitochondrial signaling pathways and associated functions for three representative model systems: mammals (*Homo sapiens, Mus musculus* and *Rattus norvegicus*), the budding yeast *S. cerevisiae* and the nematode *C. elegans*. Based on these data, we reconstructed organism-specific signaling maps for each species and overlaid them to identify shared signaling molecules, conserved pathway architectures and clade-restricted modules (Fig. 3).

**Fig. 4:**
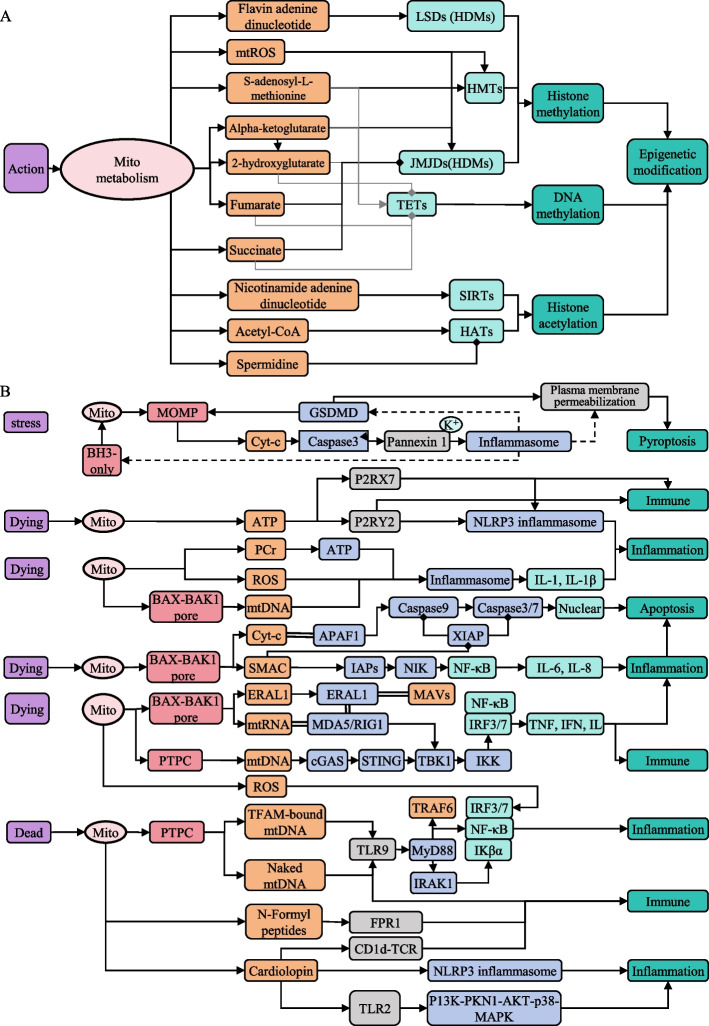
Regulatory networks of mitochondrial signaling in biological processes. **A**. Architecture of mitochondrial signaling networks modulating host epigenetic regulation. **B**. Integrated signaling networks governing cell death, immunity, and inflammation. Note: Fig. 4 share the legend with Fig. 3

**Fig. 5:**
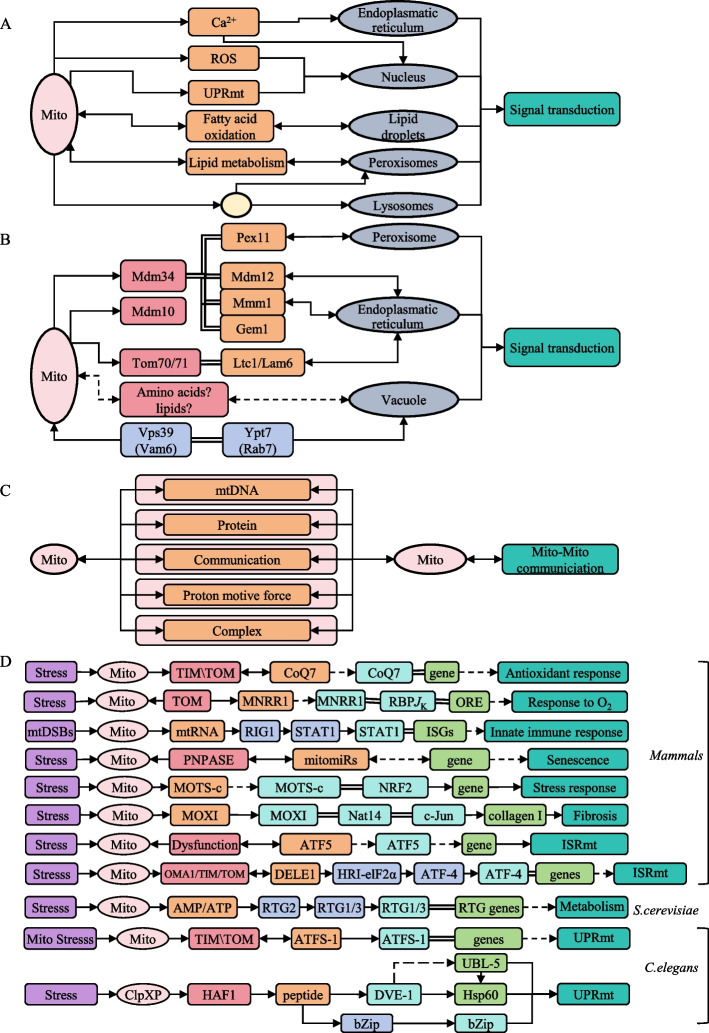
Mitochondria communicate with other organelles **A**. Interorganellar communication between mitochondria and other organelles in mammals. **B**. Contact-dependent mitochondrial-organelle networks in *S. cerevisiae*. **C**. Mitochondria-mitochondria signaling interactions. **D**. Mitochondrial-nuclear communications. Note: Fig. 5 share the legend with Fig. 3

As illustrated in Fig. 3a, a Venn diagram reveals the distribution of signaling molecules among the three taxonomic groups, revealing both evolutionarily conserved cores and lineage-specific signatures. All three groups share several central metabolites and ions, including acetyl-CoA, H_2_O_2_, methionine, nicotinamide adenine dinucleotide (NAD^+^), ROS, S-adenosylmethionine (SAM), and calcium ions (Ca^2+^). These molecules are recurrently associated with aging, cell death, oxidative signaling, and stress response regulation across species, indicating that mitochondria have retained a deeply conserved "metabolic-signaling" module since early eukaryotic evolution. For instance, mitochondrial ROS mediates mitochondria-nucleus crosstalk to modulate nuclear gene expression, while SAM couples mitochondrial one-carbon metabolism to chromatin methylation and epigenetic control. In parallel, all three groups exhibit mitochondrial retrograde mitochondria-to-nucleus signaling, mitochondrial dynamics, and regulated cell death pathways, supporting the view that stress-responsive mitochondrial-nuclear communication and quality control circuits are ancestral and highly conserved features of mitochondria. By contrast, the non-overlapping Venn sectors highlight clade-specific signal types (e.g., hormonal and cytokine-like mitokines in mammals) that likely represent later evolutionary innovations associated with multicellularity and tissue specialization.

Using apoptosis as a model process, we analyzed mitochondrial roles across these three taxonomic groups. While apoptosis is regulated by multiple factors, mitochondria and mitochondrial-associated proteins consistently play crucial roles, all involving the key signaling molecule cytochrome c (cyt-c). We constructed comparative flowcharts of apoptotic pathways in the three taxonomic groups (Fig. 3b). To aid interpretation, we defined different interaction types and related modules using distinct line styles, shapes (rectangular and circular) and color codes; this graphical convention is applied consistently across all signaling pathway diagrams in this study. In mammals, Bcl-2 family proteins modulate mitochondrial outer membrane permeability, facilitating the release of cyt-c and apoptosis-inducing factor (AIF). Cyt-c binds apoptotic protease activating factor-1 (APAF-1) to recruit caspases and execute apoptosis, while AIF translocates to the nucleus to mediate chromatin/DNA degradation. The mitochondrial dynamin-related protein (Drp1) participates in apoptosis, with its *S. cerevisiae* homolog Dnm1 and partner Fis1 similarly regulating *S. cerevisiae* apoptosis. Furthermore, both *S. cerevisiae* and *C. elegans* possess apoptosis-related homologs including AIF (apoptosis-inducing factor) and EndoG (endonuclease G) orthologs, as well as caspase-family proteins (Yca1 in *S. cerevisiae* and CED-3 in *C. elegans*) [56, 57]. Notably, *C. elegans* additionally express Bcl-2 family homologs, all of which have been functionally validated to participate in programmed cell death pathways.

Both mammals and *C. elegans* exhibit inter-tissue mitochondrial signaling with partially conserved architectures (Fig. 3c,d). In mammals, the mitokines GDF15 and FGF21 have been shown to be transcriptionally induced by ATF4 under mitochondrial stress [58]. These secreted mitokines act through specific receptors (e.g. GFRAL, FGFR1c/β-Klotho) to transmit mitochondrial stress signals to distal tissues and modulate UPRmt-related responses (Fig. 3c). In parallel, mitochondria-dependent metabolites and hormones such as adenosine, cortisol, melatonin, succinate and choline function as circulating signals that are sensed by cognate receptors in target organs and activate downstream signaling pathways (Fig. 3a,d). In *C. elegans*, an analogous inter-tissue pathway involves the Wnt ligand EGL-20 [59], the neuropeptide FLP-2 [60] and other neuropeptides, which relay mitochondrial stress signals from the nervous system to the intestine in an ATFS-1–dependent manner (Fig. 3d). Notably, serotonin acts as an evolutionarily conserved inter-tissue signal in both mammals and *C. elegans*, coupling mitochondrial stress in one tissue to responses in others. Overall, although multicellular organisms share core modules of mitochondrial intercellular communication, the diversification of tissues and cell types has been accompanied by increased evolutionary diversity in signaling molecules, receptors and downstream pathways.

### Mitochondrial signaling pathways in different functions

Mitochondria serve as pivotal signaling organelles across diverse biological processes, capable of generating outputs that either participate in single or multiple pathways, or cooperatively regulate specific processes through signal integration. Mitochondria exhibit expanded functional complexity in multicellular organisms. Our analysis specifically focuses on the metabolic regulators of epigenetic modifications [35, 61] and key mediators of immune responses [7], inflammatory processes [6], and cell death [11]. The individual signaling relationships depicted in Fig. 4 are supported by primary studies and reviews cited in MitoCommun, while we here unify them into a coherent, function-centric network view that exposes cross-talk between epigenetic regulation, cell death, immunity and inflammation.

Mitochondrial metabolites play extensive roles in regulating host epigenetics by serving as essential cofactors for epigenetic-modifying enzymes, thereby modulating broad gene expression programs. These metabolites originate from core mitochondrial pathways including the tricarboxylic acid (TCA) cycle, electron transport chain (ETC), and one-carbon metabolism, effectively coupling metabolic and genetic regulation (Fig. 4a). Our analysis revealed that these metabolites primarily regulate histone methylation/acetylation and DNA methylation by either cooperatively targeting the same enzyme or antagonistically modulating counteracting enzymes, thereby controlling transcriptional activation and silencing. For example, fumarate, succinate, α-ketoglutarate (α-KG), and 2-hydroxyglutarate (2-HG) all modulate histone/DNA demethylases, while S-adenosyl-L-methionine (SAM) serves as the essential methyl donor for histone/DNA methyltransferases. Notably, α-KG additionally regulates 2-HG levels through metabolic cross-talk. Histone methylation is primarily regulated by histone methyltransferases (HMTs) and histone demethylases (HDMs), including Lys-specific demethylases (LSDs) and Jumonji C (JmjC) domain-containing lysine demethylases (JMJDs) (Fig. 4a). DNA methylation is modulated by DNA methyltransferases (DNMTs) and ten-eleven translocation (TET) demethylases, with SAM, α-KG, 2-HG, fumarate, and succinate all targeting TET enzymes. Histone acetylation is principally controlled by histone acetyltransferases (HATs) and protein deacetylases sirtuins (SIRTs), where acetyl-CoA and spermidine regulate HATs and NAD^+^ modulates SIRTs. In Fig. 4a, we integrated data from *C. elegans* and *S. cerevisiae*: spermidine inhibits HATs activity, while mtROS activate histone methyltransferases and demethylases [35].

Mitochondria play critical regulatory roles in cell death, inflammation, and immune responses [6, 11]. Based on the curated data, we integrated these signals into a network of mitochondrial pathways that link these biological processes, as illustrated in Fig. 4b. Mitochondrial stress triggers mitochondrial outer membrane permeabilization (MOMP), releasing cytochrome c (Cyt-c) to activate both caspase-dependent apoptosis and inflammasome pathways, ultimately leading to plasma membrane rupture and pyroptosis. Mitochondria in dying cells release ATP, which binds to extracellular purinergic receptors P2RX7 and P2RY2 to initiate immune responses, while simultaneously activating the NLRP3 inflammasome to promote inflammation. Furthermore, mitochondrial DAMPs (ATP, ROS, mtDNA, and mtRNA) directly activate inflammasomes or indirectly stimulate NF-κB signaling, collectively orchestrating apoptosis, inflammatory responses, and immune activation. During cell death, BAX-BAK pores form in dying cells, releasing not only mtDNA but also SMAC and mtRNA into the cytosol to activate NF-κB signaling. The released mtRNA and mtDNA engage the TBK1-IKK-IRF3/7 pathway, thereby inducing inflammation, immune responses, and cell death. Additionally, dead cells release other forms of mtDNA including TFAM-bound mtDNA and naked mtDNA into the extracellular space, where they activate TLR9 to trigger immune and inflammatory responses in neighboring cells. The dead cells also secrete N-formyl peptides (NFPs) that bind FPR1 receptors, as well as cardiolipin that interacts with immune cell surface receptors, both initiating distinct immune and inflammatory cascades.

Collectively, our analysis shows that ROS not only participate in epigenetic regulation but also modulate diverse cellular functions through multiple pathways, including cell death, immunity, and inflammation (Fig. 4a,b). As pivotal mitochondrial redox mediators, ROS play an essential role in cellular monitoring and feedback within the mitochondrial communication network. mtDNA, in its various forms, can activate identical or distinct signaling pathways to trigger inflammatory and immune responses. Cytochrome c (Cyt c) serves as a crucial signaling molecule in both apoptosis and pyroptosis by activating downstream caspases. During cell death or under various stress conditions, mitochondrial outer membrane permeabilization (MOMP) and mitochondrial permeability transition pore (mPTP) formation lead to the exposure of mtDNA, ATP, and cardiolipin in both intracellular and extracellular compartments. These molecules bind corresponding receptors to mediate immune activation and subsequent inflammatory responses, which in turn can induce both apoptosis and pyroptosis. Mitochondrial signaling thus plays pivotal roles in cross-functional signaling networks that connect metabolism, cell death and innate immunity. Depending on the intensity, duration and subcellular localization of mitochondrial stress, the same molecular cue can elicit either adaptive, cytoprotective responses or irreversible inflammatory cell death under different conditions.

### Mitochondrial signaling pathways among different organelles

Mitochondria function as signaling organelles that not only regulate diverse biological processes across species but also serve as central hubs communicating with other cellular compartments through distinct mechanisms. These organelle-specific communication patterns vary markedly among species and tissues. In general, mitochondria establish frequent signaling crosstalk with other organelles through a combination of metabolite exchange and the formation of physical contact platforms. Building on published studies of organelle contact sites and mitochondrial-nuclear signaling [14, 32, 36, 62, 63], we integrated these dispersed findings into a unified, multi-organellar framework. This integration is summarized in Fig. 5, which delineates core mitochondria-organelle communication routes and highlights both conserved and system-specific interaction patterns.

In mammalian cells, ER interact intimately through mitochondria-associated ER membranes (MAMs), which facilitate calcium and ROS signaling and coordinately regulate the UPRmt via mitochondrial-nuclear communication. In addition, mitochondria exchange fatty acids with lipid droplets, swap lipid metabolites with peroxisomes and lysosomes, and deliver bulk cargo to these organelles via mitochondria-derived vesicles (Fig. 5a).

In *S. cerevisiae*, mitochondria also communicate with other organelles via membrane contact sites, where specialized membrane proteins establish physical platforms to facilitate metabolite exchange and functional coordination. For example, mitochondrial outer membrane proteins such as Mdm34 and Tom70 mediate contacts with peroxisomes, the ER and vacuoles, enabling dynamic metabolic exchange, energy transfer, and potential macromolecular protein transport. Notably, mitochondria engage in extensive material and signal exchange with vacuoles, likely involving amino acids, lipids, and other metabolites (Fig. 5b).

In mammalian systems, mitochondria exhibit pronounced tissue-specific differences in morphology and abundance. Inter-mitochondrial communication typically occurs through kissing junctions, nanotunnels, and membrane fusion events, facilitating the exchange and integration of macromolecules (mtDNA and proteins), membrane components, and mitochondrial membrane potential (MMP) signaling (Fig. 5c). In tissues with densely packed, mitochondria-rich cells, these organelles can even transmit electrical signals over relatively long distances, and mitochondria from adjacent cells may communicate via intercellular nanotunnels.

Mitochondrial-nuclear communication plays pivotal roles in diverse biological processes through both anterograde and retrograde signaling pathways. Nuclear-encoded mitochondrial proteins, such as coenzyme Q7 (CoQ7), mitochondrial nuclear retrograde regulator 1 (MNRR1) and the metabolic enzyme hexokinase 2 (HK2) act as bidirectional signaling molecules that normally function in mitochondria but can translocate to the nucleus under specific conditions to regulate nuclear gene expression. Furthermore, EndoG, Drp1, and Bcl-2 can also serve as nuclear regulatory factors in defined contexts. This inter-organelle communication additionally involves the bidirectional exchange of nucleic acids. Mitochondria-derived nucleic acids can be transported to the nucleus, where they participate in gene regulation. For example, Mitochondrial DNA double-strand breaks (mtDSBs) induce herniation of the mitochondrial membrane, leading to the release of mtRNA that is detected by the cytoplasmic RNA sensor RIG-I. This recognition triggers STAT1 nuclear translocation and subsequent upregulation of interferon-stimulated genes (ISGs), thereby modulating innate immune responses [64]. Moreover, nucleus-encoded mitochondria-localized microRNAs (mitomiRs) serve as bidirectional communicators between the nuclear and mitochondrial compartments and have been implicated in the control of cellular senescence [65]. Figure 5d further illustrates that mitochondrial-derived peptides can also translocate into the nucleus. Under stress conditions, the peptide MOTS-c accumulates in the nucleus, where it binds stress-responsive DNA elements and transcriptional regulators such as nuclear factor erythroid 2-related factor 2 (NRF2) to modulate stress-induced gene expression [66]. With persistent profibrotic stress, the mitochondrial micropeptide MOXI (Mitoregulin) becomes phosphorylated and accumulates in the nucleus, where it enhances collagen I gene transcription and promotes fibrosis [67].

We next consider transcription factor pathways that mediate mitochondrial stress responses in three taxonomic groups: ATF4 (Activating Transcription Factor 4) and ATF5 in mammals, ATFS-1 in *C. elegans* and the RTG2-RTG1/3 pathways in *S. cerevisiae*. As summarized in Fig. 5d, mitochondrial stress signals drive retrograde signaling and nuclear translocation of these factors, which then activate stress-responsive genes to alleviate mitochondrial dysfunction. In mammals, mitochondrial protein import defects cause the normally mitochondria-localized protein DELE1 to accumulate in the cytosol, where it activates the integrated stress response (ISR) kinase HRI and promotes phosphorylation of eIF2α, thereby increasing translation of ATF4, CHOP and ATF5. These transcription factors, in turn, induce expression of stress-responsive genes to coordinate the mitochondrial integrated stress response (ISRmt) [68]. In *S. cerevisiae*, mitochondrial retrograde (RTG) signaling is activated when RTG2 senses dysfunction signals such as altered AMP/ATP ratios under nutritional or metabolic stress. RTG2 activation triggers nuclear translocation of the transcription factors RTG1 and RTG3, which then reprogram metabolic gene expression through retrograde signaling [69] (Fig. 5d). In *C. elegans*, ATFS-1 is a transcription factor with both mitochondrial and nuclear localization signals: under basal conditions it is imported into mitochondria and degraded, whereas impaired mitochondrial import causes ATFS-1 to accumulate in the cytosol, translocate to the nucleus and induce UPRmt target genes such as *HSP60* [70, 71] (Fig. 5d). Collectively, these examples illustrate a conserved principle whereby mitochondrial dysfunction promotes the nuclear translocation of transcription factors. In S. cerevisiae, the retrograde signaling pathway is relatively simple, whereas in C. elegans the transcription factor ATFS-1 is considered a functional ortholog of mammalian ATF5; in both systems, defects in mitochondrial protein import activate these factors, promote their nuclear accumulation and induce expression of UPRmt target genes such as HSP60. By contrast, mammalian cells exhibit additional regulatory layers and pronounced tissue specificity in mito–nuclear communication.

In summary, our systematic analysis reveals a conserved mitochondrial signaling paradigm in which, under basal or stimulus-specific conditions, mitochondria release distinct molecular signals that target single or multiple effectors to initiate downstream pathways. These signals orchestrate epigenetic remodeling, nuclear responses, and transcriptional/translational regulation, ultimately coordinating mitochondrial and cellular homeostasis through integrated signaling networks. The comprehensive MitoCommun database (http://mitocommun.qscn.online/) provides additional details on these regulatory mechanisms and their functional consequences.

## Discussion

The mitochondrial communication database and network established in this study contribute to elucidating the fundamental architecture of mitochondrial signaling networks. Through systematic analysis, we demonstrate that mitochondrial signal transduction exhibits complex cross-network properties integrating multiple signal types, where individual signaling molecules can participate (engage) in distinct pathways and functional regulations under diverse stress responses and cellular states. Mitochondria function not only as signal emitters but also as signal sensors and integrators. However, numerous aspects of these sophisticated and microscale mitochondrial activities remain unexplored, requiring deeper investigation at higher resolution. Recent advances in subcellular research have spawned new disciplines such as subcellular proteomics and metabolomics to address these questions[72]. Despite being extensively studied, mitochondria engage in complex interactions with other organelles, with current research primarily focusing on their communications with the endoplasmic reticulum and nucleus. The involvement of diverse proteins and nucleic acids in these processes presents substantial experimental challenges. The mitochondrial genome contains differentially methylated regions (mtDMRs) which vary in response to stress, enriched with recognition sites for nuclear-derived DNA-binding factors (such as ATF4), revealing a new layer of mitochondrial-nuclear interaction[73]. Investigating these processes presents several fundamental experimental challenges, including the need to precisely resolve protein subcellular localization, conformational states, and dynamic behaviors [3, 74]. It also requires reliable methods to identify and functionally validate peptide-derived signaling mediators [67, 75] and comprehensively characterize diverse nucleic acid components, particularly small regulatory RNAs and mitochondrial DNA fragments [65, 67]. As research progresses to deeper levels, experimental complexity and workload increase substantially. Furthermore, numerous unexplored/unidentified sequences await systematic scientific exploration and validation[3, 76].

From a methodological perspective, our study has several limitations. First, despite systematic searches in PubMed and Web of Science and iterative citation chasing from recent high-impact reviews, we cannot claim to have captured all mitochondrial communication studies published to date. The concept of "mitochondrial signaling" has only been formalized and systematically discussed in the last decade[14, 72], and many earlier studies are embedded in disease- or pathway-specific literature without explicit reference to communication or signaling terminology. Consequently, some relevant pathways may have been missed, particularly those described in older or less accessible journals. Second, the mammalian component of MitoCommun is dominated by data from humans, mice and rats, which we aggregated into a single "mammalian" group for most analyses. Although this is supported by the strong conservation of core mitochondrial pathways, it inevitably obscures species-specific physiological, metabolic and circadian adaptations that will need to be addressed in future species-resolved comparisons. Finally, because all curation steps were performed manually, the database is likely biased toward well-studied pathways and model systems. We therefore regard MitoCommun as a high-confidence but incomplete resource that will require ongoing community-driven updates as new data become available.

The current mitochondrial communication network, while providing valuable insights, remains incomplete with significant knowledge gaps and undefined nodes (as highlighted in Figs. 3 and 5). To address these limitations, future research should implement integrated multi-omics strategies to construct more comprehensive pathways and the corresponding communication networks. At the genomic level, future research should prioritize systematic annotation and functional validation of mitochondrial activity-related genes, particularly those involved in mitochondrial-nuclear and inter-organellar communication. It is very important to decipher gene–gene interaction networks to elucidate the complex regulatory relationships governing mitochondrial functions. Transcriptomic investigations require focused efforts to decode mitochondrial-associated regulatory networks underlying diverse functional phenotypes, with special emphasis on characterizing the mechanistic roles of small RNAs (sRNAs) in mitochondrial communication pathways. From a proteomic perspective, systematic identification of novel mitochondrial-localized proteins and new functional characterization of known proteins may reveal previously unrecognized mitochondrial-related functions, thereby expanding the signaling network. Metabolomic approaches should be conducted to not only uncover non-canonical roles of characterized mitochondrial metabolites [5, 77–79], but also identify novel metabolic intermediates through comprehensive pathway analysis to complete the metabolic network architecture [3].

In addition, species-specific differences in whole-body metabolism, lifestyle and circadian behaviour are likely to shape mitochondrial signaling networks in ways that are not apparent from our aggregated analyses [80–82]. For example, nocturnal rodents and diurnal humans differ markedly in feeding-fasting patterns, tissue-specific energy demands and exposure to environmental stressors, all of which influence mitochondrial morphology, dynamics and metabolite pools [80, 83]. These differences may change how otherwise conserved signaling modules are used in quantitative terms—for instance, by shifting the balance between hormonal and metabolite-based communication or by altering the tissue origin and systemic impact of mitokines [58, 84]. Future work that combines species-resolved MitoCommun annotations with time-resolved multi-omics datasets should help to resolve this context-dependent rewiring of conserved mitochondrial signaling circuits [85].

Mitochondria, serving as a critical evolutionary link between bacteria and eukaryotes, mediate cross-domain communication between microbes and eukaryotic cells, offering novel insights into the molecular dialogue underlying endosymbiotic origins [86]. As a highly conserved organelle in eukaryotes, mitochondria play a pivotal role as communication hubs during fungal-host interactions. They retain bacterial-origin signaling pathways that establish cross-domain crosstalk with host organelles, thereby forming a tripartite communication network connecting bacteria, fungi, and host cells [87, 88]. From the bacterial perspective, microbial metabolites (e.g., short-chain fatty acids) modulate host immune homeostasis by regulating mitochondrial function. Bacterial metabolites (Colanic acid) regulate host mitochondrial dynamics and the UPRmt to promote longevity [87]. The N-(3-oxododecanoyl)-homoserine lactone (C12) produced by *Pseudomonas aeruginosa* induces mitochondrial membrane depolarization, cytochrome c release, and caspase-dependent apoptosis in host cells [89]. Fungal mitochondria, conversely, employ retrograde signaling (RTG pathway) to control expression of bacterial competition-related genes. Host cells maintain this tripartite equilibrium through selective mitophagy of damaged mitochondria [90]. This sophisticated communication network is particularly prominent in inflammatory bowel diseases, where mitochondrial DNA release acts as damage-associated molecular patterns (DAMPs) that undergo microbial methylation before being re-recognized by host receptors, establishing an exquisite feedback regulatory loop [91, 92]. Notably, the evolutionary retention of bacterial-origin communication mechanisms, such as quorum sensing systems [93–96], in mitochondria represents an emerging research frontier, as these systems may coordinate both mitochondrial phenotypic plasticity and tripartite interactions. While further refinement is ongoing, MitoCommun constitutes a unique knowledge base that systematically characterizes intercellular communication pathways, offering mechanistic insights into the molecular legacy of endosymbiotic evolution. Taken together, our comparative analysis across yeast, nematodes and mammals outlines a mechanistic and evolutionary basis for identifying mitochondrial signaling nodes as potential drug targets. By bringing together conserved communication modules and mitokines involved in stress responses, cell death and metabolic control, MitoCommun can support hypothesis-driven searches for therapeutic strategies for mitochondrial diseases and related disorders. More broadly, this resource refines our understanding of mitochondrial evolutionary biology and may help to guide the design of mitochondria-targeted interventions.

In conclusion, this study comprehensively curated extensive data on mitochondrial communication, leading to the development of a user-friendly database that facilitates efficient browsing and search of results. We have integrated multiple mitochondrial signals and pathways, incorporating detailed cellular stress responses, to construct robust mitochondrial signaling networks. These networks span diverse organisms, organelles, and functional processes, providing a valuable resource for further research into mitochondrial function and interorganellar communication.

## Supplementary Information

Additional file 1.

## Data Availability

MitoCommun is freely available at: [http://mitocommun.qscn.online/](http:/mitocommun.qscn.online). We will continuously update the database. The data are available from the corresponding author upon reasonable request via email. In addition, Additional file 1 (Excel spreadsheet) containing all curated references is provided with this article.

## References

[1] Eisner V, Picard M, Hajnoczky G. Mitochondrial dynamics in adaptive and maladaptive cellular stress responses. Nat Cell Biol. 2018;20(7):755–65. 29950571 10.1038/s41556-018-0133-0 PMC6716149

[2] Rinschen MM, Ivanisevic J, Giera M, Siuzdak G. Identification of bioactive metabolites using activity metabolomics. Nat Rev Mol Cell Biol. 2019;20(6):353–67. 30814649 10.1038/s41580-019-0108-4 PMC6613555

[3] Guo Y, Wen H, Chen Z, Jiao M, Zhang Y, Ge D, Liu R, Gu J. Conjoint analysis of succinylome and phosphorylome reveals imbalanced HDAC phosphorylation-driven succinylayion dynamic contibutes to lung cancer. Brief Bioinform. 2024;25(5):bbae415. 10.1093/bib/bbae415 PMC11343571 39179249

[4] Chakrabarty RP, Chandel NS. Mitochondria as signaling organelles control mammalian stem cell fate. Cell Stem Cell. 2021;28(3):394–408. 33667360 10.1016/j.stem.2021.02.011 PMC7944920

[5] Kafkia E, Andres-Pons A, Ganter K, Seiler M, Smith TS, Andrejeva A, et al. Operation of a TCA cycle subnetwork in the mammalian nucleus. Sci Adv. 2022;8(35):eabq5206. 36044572 10.1126/sciadv.abq5206 PMC9432838

[6] Marchi S, Guilbaud E, Tait SWG, Yamazaki T, Galluzzi L. Mitochondrial control of inflammation. Nat Rev Immunol. 2023;23(3):159–73. 35879417 10.1038/s41577-022-00760-x PMC9310369

[7] Banoth B, Cassel SL. Mitochondria in innate immune signaling. Transl Res. 2018;202:52–68. 30165038 10.1016/j.trsl.2018.07.014 PMC6218307

[8] Collier JJ, Olahova M, McWilliams TG, Taylor RW. Mitochondrial signalling and homeostasis: from cell biology to neurological disease. Trends Neurosci. 2023;46(2):137–52. 36635110 10.1016/j.tins.2022.12.001

[9] Ma T, Zeng X, Liu M, Xu S, Wang Y, Wu Q, et al. Analysis and identification of mitochondria-related genes associated with age-related hearing loss. BMC Genomics. 2025;26(1):218. 40045222 10.1186/s12864-025-11287-5 PMC11881475

[10] Tigano M, Vargas DC, Tremblay-Belzile S, Fu Y, Sfeir A. Nuclear sensing of breaks in mitochondrial DNA enhances immune surveillance. Nature. 2021;591(7850):477–81. 33627873 10.1038/s41586-021-03269-w

[11] Bock FJ, Tait SWG. Mitochondria as multifaceted regulators of cell death. Nat Rev Mol Cell Biol. 2020;21(2):85–100. 31636403 10.1038/s41580-019-0173-8

[12] Cartes-Saavedra B, Ghosh A, Hajnóczky G. The roles of mitochondria in global and local intracellular calcium signalling. Nat Rev Mol Cell Biol. 2025;26(6):456–75. 39870977 10.1038/s41580-024-00820-1

[13] Suomalainen A, Nunnari J. Mitochondria at the crossroads of health and disease. Cell. 2024;187(11):2601–27. 38788685 10.1016/j.cell.2024.04.037

[14] Picard M, Shirihai OS. Mitochondrial signal transduction. Cell Metab. 2022;34(11):1620–53. 36323233 10.1016/j.cmet.2022.10.008 PMC9692202

[15] Zhang H, Li X, Fan W, Pandovski S, Tian Y, Dillin A. Inter-tissue communication of mitochondrial stress and metabolic health. Life Metab. 2023;2(1):load001. 10.1093/lifemeta/load001 PMC10399134 37538245

[16] Wang YP, Sharda A, Xu SN, van Gastel N, Man CH, Choi U, et al. Malic enzyme 2 connects the Krebs cycle intermediate fumarate to mitochondrial biogenesis. Cell Metab. 2021;33(5):1027-1041 e1028. 33770508 10.1016/j.cmet.2021.03.003 PMC10472834

[17] Liu Y, Liu S, Tomar A, Yen FS, Unlu G, Ropek N, et al. Autoregulatory control of mitochondrial glutathione homeostasis. Science. 2023;382(6672):820–8. 37917749 10.1126/science.adf4154 PMC11170550

[18] Ryu KW, Fung TS, Baker DC, Saoi M, Park J, Febres-Aldana CA, et al. Cellular ATP demand creates metabolically distinct subpopulations of mitochondria. Nature. 2024;635(8039):746–54. 39506109 10.1038/s41586-024-08146-w PMC11869630

[19] Zhou H, Ren J, Toan S, Mui D. Role of mitochondrial quality surveillance in myocardial infarction: from bench to bedside. Ageing Res Rev. 2021;66:101250. 33388396 10.1016/j.arr.2020.101250

[20] Hill S, Sataranatarajan K, Van Remmen H. Role of signaling molecules in mitochondrial stress response. Front Genet. 2018;9:225. 30042784 10.3389/fgene.2018.00225 PMC6048194

[21] Di Mambro T, Pellielo G, Agyapong ED, Carinci M, Chianese D, Giorgi C, Morciano G, Patergnani S, Pinton P, Rimessi A. The tricky connection between extracellular vesicles and mitochondria in inflammatory-related diseases. Int J Mol Sci. 2023;24(9):8181. 10.3390/ijms24098181. 10.3390/ijms24098181 PMC10179665 37175888

[22] Angajala A, Lim S, Phillips JB, Kim JH, Yates C, You Z, et al. Diverse roles of mitochondria in immune responses: novel insights into immuno-metabolism. Front Immunol. 2018;9:1605. 30050539 10.3389/fimmu.2018.01605 PMC6052888

[23] Shimura T. Mitochondrial signaling pathways associated with DNA damage responses. Int J Mol Sci. 2023;24(7):6128. 10.3390/ijms24076128 PMC10094106 37047099

[24] Yun J, Finkel T. Mitohormesis. Cell Metab. 2014;19(5):757–66. 24561260 10.1016/j.cmet.2014.01.011 PMC4016106

[25] Bohovych I, Khalimonchuk O. Sending out an SOS: mitochondria as a signaling hub. Front Cell Dev Biol. 2016;4:109. 27790613 10.3389/fcell.2016.00109 PMC5061732

[26] Chandel NS. Mitochondria as signaling organelles. BMC Biol. 2014;12:34. 24884669 10.1186/1741-7007-12-34 PMC4035690

[27] Picard M, McEwen BS, Epel ES, Sandi C. An energetic view of stress: focus on mitochondria. Front Neuroendocrinol. 2018;49:72–85. 29339091 10.1016/j.yfrne.2018.01.001 PMC5964020

[28] Chakrabarty RP, Chandel NS. Beyond ATP, new roles of mitochondria. Biochemist. 2022;44(4):2–8. 36248614 10.1042/bio_2022_119 PMC9558425

[29] Konig T, McBride HM. Mitochondrial-derived vesicles in metabolism, disease, and aging. Cell Metab. 2024;36(1):21–35. 38171335 10.1016/j.cmet.2023.11.014

[30] Hazan Ben-Menachem R, Lintzer D, Ziv T, Das K, Rosenhek-Goldian I, Porat Z, et al. Mitochondrial-derived vesicles retain membrane potential and contain a functional ATP synthase. EMBO Rep. 2023;24(5):e56114. 36929726 10.15252/embr.202256114 PMC10157309

[31] Huang X, Sun L, Ji S, Zhao T, Zhang W, Xu J, et al. Kissing and nanotunneling mediate intermitochondrial communication in the heart. Proc Natl Acad Sci U S A. 2013;110(8):2846–51. 23386722 10.1073/pnas.1300741110 PMC3581932

[32] Boardman NT, Trani G, Scalabrin M, Romanello V, Wust RCI. Intracellular to interorgan mitochondrial communication in striated muscle in health and disease. Endocr Rev. 2023;44(4):668–92. 36725366 10.1210/endrev/bnad004 PMC10335175

[33] Dakik P, Titorenko VI. Communications between mitochondria, the nucleus, vacuoles, peroxisomes, the endoplasmic reticulum, the plasma membrane, lipid droplets, and the cytosol during yeast chronological aging. Front Genet. 2016;7:177. 27729926 10.3389/fgene.2016.00177 PMC5037234

[34] Hijazi I, Knupp J, Chang A. Retrograde signaling mediates an adaptive survival response to endoplasmic reticulum stress in Saccharomyces cerevisiae. J Cell Sci. 2020;133(6):jcs241539. 10.1242/jcs.241539 PMC7132770 32005698

[35] Zhu D, Li X, Tian Y. Mitochondrial-to-nuclear communication in aging: an epigenetic perspective. Trends Biochem Sci. 2022;47(8):645–59. 35397926 10.1016/j.tibs.2022.03.008

[36] Ryan MT, Hoogenraad NJ. Mitochondrial-nuclear communications. Annu Rev Biochem. 2007;76:701–22. 17227225 10.1146/annurev.biochem.76.052305.091720

[37] Druseikis ME, Covo S. Synthetic lethality between toxic amino acids, RTG-target genes and chaperones in Saccharomyces cerevisiae. Yeast. 2024;41(9):549–59. 39078098 10.1002/yea.3975

[38] Liu Y, Zhou J, Zhang N, Wu X, Zhang Q, Zhang W, et al. Two sensory neurons coordinate the systemic mitochondrial stress response via GPCR signaling in C. elegans. Dev Cell. 2022;57(21):2469-2482 e2465. 36309009 10.1016/j.devcel.2022.10.001

[39] Shao LW, Niu R, Liu Y. Neuropeptide signals cell non-autonomous mitochondrial unfolded protein response. Cell Res. 2016;26(11):1182–96. 27767096 10.1038/cr.2016.118 PMC5099867

[40] Stefano GB, Buttiker P, Weissenberger S, Esch T, Anders M, Raboch J, et al. Independent and sensory human mitochondrial functions reflecting symbiotic evolution. Front Cell Infect Microbiol. 2023;13:1130197. 37389212 10.3389/fcimb.2023.1130197 PMC10302212

[41] Gu Z, Li J, Gao S, Gong M, Wang J, Xu H, et al. Intermitobase: an annotated database and analysis platform of protein-protein interactions for human mitochondria. BMC Genomics. 2011;12(1):335. 21718467 10.1186/1471-2164-12-335 PMC3142533

[42] Kogelnik AM, Lott MT, Brown MD, Navathe SB, Wallace DC. MITOMAP: a human mitochondrial genome database–1998 update. Nucleic Acids Res. 1998;26(1):112–5. 9399813 10.1093/nar/26.1.112 PMC147233

[43] Rath S, Sharma R, Gupta R, Ast T, Chan C, Durham TJ, et al. MitoCarta3.0: an updated mitochondrial proteome now with sub-organelle localization and pathway annotations. Nucleic Acids Res. 2021;49(D1):D1541–7. 33174596 10.1093/nar/gkaa1011 PMC7778944

[44] Wishart DS, Guo A, Oler E, Wang F, Anjum A, Peters H, et al. HMDB 5.0: the human metabolome database for 2022. Nucleic Acids Res. 2022;50(D1):D622-d631. 34986597 10.1093/nar/gkab1062 PMC8728138

[45] Noronha A, Modamio J, Jarosz Y, Guerard E, Sompairac N, Preciat G, et al. The virtual metabolic human database: integrating human and gut microbiome metabolism with nutrition and disease. Nucleic Acids Res. 2019;47(D1):D614-d624. 30371894 10.1093/nar/gky992 PMC6323901

[46] Ramirez-Gaona M, Marcu A, Pon A, Guo AC, Sajed T, Wishart NA, et al. YMDB 2.0: a significantly expanded version of the yeast metabolome database. Nucleic Acids Res. 2017;45(D1):D440-d445. 27899612 10.1093/nar/gkw1058 PMC5210545

[47] Kanehisa M, Furumichi M, Tanabe M, Sato Y, Morishima K. KEGG: new perspectives on genomes, pathways, diseases and drugs. Nucleic Acids Res. 2017;45(D1):D353-d361. 27899662 10.1093/nar/gkw1092 PMC5210567

[48] Lagies S, Pan D, Mohl DA, Plattner DA, Gentle IE, Kammerer B. Mitochondrial metabolomics of Sym1-Depleted yeast cells revealed them to be lysine auxotroph. Cells. 2023;12(5):692. 10.3390/cells12050692 PMC10000845 36899826

[49] Pan D, Wiedemann N, Kammerer B. Heat stress-induced metabolic remodeling in saccharomyces cerevisiae. Metabolites. 2019;9(11):266. 10.3390/metabo9110266 PMC6918159 31694329

[50] Pan D, Lindau C, Lagies S, Wiedemann N, Kammerer B. Metabolic profiling of isolated mitochondria and cytoplasm reveals compartment-specific metabolic responses. Metabolomics. 2018;14(5):59. 29628813 10.1007/s11306-018-1352-x PMC5878833

[51] UniProt. the Universal Protein Knowledgebase in 2023. Nucleic Acids Res. 2023;51(D1):D523-d531. 36408920 10.1093/nar/gkac1052 PMC9825514

[52] Kaur D, Arora A, Patiyal S, Raghava GPS. Hmrbase2: a comprehensive database of hormones and their receptors. Hormones Athens. 2023;22(3):359–66. 37291365 10.1007/s42000-023-00455-5

[53] Szklarczyk D, Gable AL, Lyon D, Junge A, Wyder S, Huerta-Cepas J, et al. STRING v11: protein-protein association networks with increased coverage, supporting functional discovery in genome-wide experimental datasets. Nucleic Acids Res. 2019;47(D1):D607-d613. 30476243 10.1093/nar/gky1131 PMC6323986

[54] Oughtred R, Stark C, Breitkreutz BJ, Rust J, Boucher L, Chang C, et al. The BioGRID interaction database: 2019 update. Nucleic Acids Res. 2019;47(D1):D529-d541. 30476227 10.1093/nar/gky1079 PMC6324058

[55] Gilson MK, Liu T, Baitaluk M, Nicola G, Hwang L, Chong J. BindingDB in 2015: a public database for medicinal chemistry, computational chemistry and systems pharmacology. Nucleic Acids Res. 2016;44(D1):D1045-1053. 26481362 10.1093/nar/gkv1072 PMC4702793

[56] Guaragnella N, Zdralevic M, Antonacci L, Passarella S, Marra E, Giannattasio S. The role of mitochondria in yeast programmed cell death. Front Oncol. 2012;2:70. 22783546 10.3389/fonc.2012.00070 PMC3388595

[57] Wang X, Yang C, Chai J, Shi Y, Xue D. Mechanisms of AIF-mediated apoptotic DNA degradation in *Caenorhabditis elegans*. Science. 2002;298(5598):1587–92. 12446902 10.1126/science.1076194

[58] Kang SG, Choi MJ, Jung SB, Chung HK, Chang JY, Kim JT, et al. Differential roles of GDF15 and FGF21 in systemic metabolic adaptation to the mitochondrial integrated stress response. iScience. 2021;24(3):102181. 33718833 10.1016/j.isci.2021.102181 PMC7920832

[59] Zhang Q, Wu X, Chen P, Liu L, Xin N, Tian Y, et al. The mitochondrial unfolded protein response is mediated cell-non-autonomously by Retromer-Dependent Wnt signaling. Cell. 2018;174(4):870–83. 30057120 10.1016/j.cell.2018.06.029 PMC6086732

[60] Deng P, Haynes CM. The mitokine quest(ion). Cell Res. 2016;26(12):1265–6. 27886169 10.1038/cr.2016.138 PMC5143424

[61] Mentch SJ, Locasale JW. One-carbon metabolism and epigenetics: understanding the specificity. Ann N Y Acad Sci. 2016;1363(1):91–8. 26647078 10.1111/nyas.12956 PMC4801744

[62] Murley A, Nunnari J. The emerging network of mitochondria-organelle contacts. Mol Cell. 2016;61(5):648–53. 26942669 10.1016/j.molcel.2016.01.031 PMC5554544

[63] Gonzalez-Arzola K, Diaz-Quintana A. Mitochondrial factors in the cell nucleus. Int J Mol Sci. 2023;24(17):13656. 10.3390/ijms241713656 PMC10563088 37686461

[64] Rigon M, Townley AR, Campanella M. Mitochondria ensure immune surveillance by retro-communication with the nucleus. Cell Metab. 2021;33(5):853–5. 33951470 10.1016/j.cmet.2021.04.013

[65] Giordani C, Silvestrini A, Giuliani A, Olivieri F, Rippo MR. Micrornas as factors in bidirectional crosstalk between Mitochondria and the nucleus during cellular senescence. Front Physiol. 2021;12:734976. 34566699 10.3389/fphys.2021.734976 PMC8458936

[66] Yong CQY, Tang BL. A mitochondrial encoded messenger at the nucleus. Cells. 2018;7(8):105. 10.3390/cells7080105 PMC6115982 30104535

[67] Cai J, Dong Z. Two-way communication between the nucleus and mitochondria via a micropeptide in renal fibrosis. Kidney Int. 2023;103(5):833–5. 37085255 10.1016/j.kint.2023.02.009

[68] Guo XY, Aviles G, Liu Y, Tian RL, Unger BA, Lin YHT, et al. Mitochondrial stress is relayed to the cytosol by an OMA1-DELE1-HRI pathway. Nature. 2020;579(7799):427-+. 32132707 10.1038/s41586-020-2078-2 PMC7147832

[69] Trendeleva TA, Zvyagilskaya RA. Retrograde signaling as a mechanism of yeast adaptation to unfavorable factors. Biochemistry (Mosc). 2018;83(2):98–106. 29618296 10.1134/S0006297918020025

[70] Haynes CM, Petrova K, Benedetti C, Yang Y, Ron D. ClpP mediates activation of a mitochondrial unfolded protein response in C. elegans. Dev Cell. 2007;13(4):467–80. 17925224 10.1016/j.devcel.2007.07.016

[71] Haynes CM, Yang Y, Blais SP, Neubert TA, Ron D. The matrix peptide exporter HAF-1 signals a mitochondrial UPR by activating the transcription factor ZC376.7 in *C. elegans*. Mol Cell. 2010;37(4):529–40. 20188671 10.1016/j.molcel.2010.01.015 PMC2846537

[72] Monzel AS, Enriquez JA, Picard M. Multifaceted mitochondria: moving mitochondrial science beyond function and dysfunction. Nat Metab. 2023;5(4):546–62. 37100996 10.1038/s42255-023-00783-1 PMC10427836

[73] Lees J, Pertille F, Lotvedt P, Jensen P, Bosagna CG. The mitoepigenome responds to stress, suggesting novel mito-nuclear interactions in vertebrates. BMC Genomics. 2023;24(1):561. 37736707 10.1186/s12864-023-09668-9 PMC10515078

[74] Vogtle FN, Burkhart JM, Gonczarowska-Jorge H, Kucukkose C, Taskin AA, Kopczynski D, et al. Landscape of submitochondrial protein distribution. Nat Commun. 2017;8(1):290. 28819139 10.1038/s41467-017-00359-0 PMC5561175

[75] Merry TL, Chan A, Woodhead JST, Reynolds JC, Kumagai H, Kim SJ, et al. Mitochondrial-derived peptides in energy metabolism. Am J Physiol Endocrinol Metab. 2020;319(4):E659–66. 32776825 10.1152/ajpendo.00249.2020 PMC7750512

[76] Abdellah M, Cantero JJG, Guerrero NR, Foni A, Coggan JS, Cali C, Agus M, Zisis E, Keller D, Hadwiger M, et al. Ultraliser: a framework for creating multiscale, high-fidelity and geometrically realistic 3D models for in silico neuroscience. Brief Bioinform. 2023;24(1):bbac491. 10.1093/bib/bbac491 PMC9851302 36434788

[77] Li Q, Hoppe T. Role of amino acid metabolism in mitochondrial homeostasis. Front Cell Dev Biol. 2023;11:1127618. 36923249 10.3389/fcell.2023.1127618 PMC10008872

[78] Toyokawa Y, Koonthongkaew J, Takagi H. An overview of branched-chain amino acid aminotransferases: functional differences between mitochondrial and cytosolic isozymes in yeast and human. Appl Microbiol Biotechnol. 2021;105(21–22):8059–72. 34622336 10.1007/s00253-021-11612-4

[79] Fernandez-Veledo S, Ceperuelo-Mallafre V, Vendrell J. Rethinking succinate: an unexpected hormone-like metabolite in energy homeostasis. Trends Endocrinol Metab. 2021;32(9):680–92. 34301438 10.1016/j.tem.2021.06.003

[80] Pickel L, Sung HK. Feeding rhythms and the circadian regulation of metabolism. Front Nutr. 2020;7:39. 32363197 10.3389/fnut.2020.00039 PMC7182033

[81] Jiang Y, Shi J, Tai J, Yan L. Circadian regulation in diurnal mammals: neural mechanisms and implications in translational research. Biology (Basel). 2024;13(12):958. 10.3390/biology13120958 PMC11727363 39765625

[82] Ezagouri S, Asher G. Circadian control of mitochondrial dynamics and functions. Curr Opin Physiol. 2018;5:25–9.

[83] Storoschuk KL, Lesiuk D, Nuttall J, LeBouedec M, Khansari A, Islam H, et al. Impact of fasting on the AMPK and PGC-1alpha axis in rodent and human skeletal muscle: a systematic review. Metabolism. 2024;152:155768. 38154612 10.1016/j.metabol.2023.155768

[84] Jena J, Garcia-Pena LM, Pereira RO. The roles of FGF21 and GDF15 in mediating the mitochondrial integrated stress response. Front Endocrinol (Lausanne). 2023;14:1264530. 37818094 10.3389/fendo.2023.1264530 PMC10561105

[85] Kelley LP, Hu SH, Boswell SA, Sorger PK, Ringel AE, Haigis MC. Integrated analysis of transcriptional and metabolic responses to mitochondrial stress. Cell Rep Methods. 2025;5(4):101027. 40233762 10.1016/j.crmeth.2025.101027 PMC12256945

[86] Han B, Lin CJ, Hu G, Wang MC. Inside out’- a dialogue between mitochondria and bacteria. FEBS J. 2019;286(4):630–41. 30390412 10.1111/febs.14692 PMC6613823

[87] Han B, Sivaramakrishnan P, Lin CJ, Neve IAA, He J, Tay LWR, et al. Microbial genetic composition tunes host longevity. Cell. 2017;169(7):1249-1262 e1213. 28622510 10.1016/j.cell.2017.05.036 PMC5635830

[88] Wu S, Bu X, Chen D, Wu X, Wu H, Caiyin Q, Qiao J. Molecules-mediated bidirectional interactions between microbes and human cells. npj Biofilms and Microbiomes. 2025;11(1):38. 10.1038/s41522-025-00657-2 PMC11880406 40038292

[89] Schwarzer C, Fu Z, Morita T, Whitt AG, Neely AM, Li C, et al. Paraoxonase 2 serves a proapopotic function in mouse and human cells in response to the *Pseudomonas aeruginosa* quorum-sensing molecule N-(3-oxododecanoyl)-homoserine lactone. J Biol Chem. 2015;290(11):7247–58. 25627690 10.1074/jbc.M114.620039 PMC4358143

[90] Li M, Wu L, Si H, Wu Y, Liu Y, Zeng Y, et al. Engineered mitochondria in diseases: mechanisms, strategies, and applications. Signal Transduct Target Ther. 2025;10(1):71. 40025039 10.1038/s41392-024-02081-y PMC11873319

[91] Jackson DN, Theiss AL. Gut bacteria signaling to mitochondria in intestinal inflammation and cancer. Gut Microbes. 2020;11(3):285–304. 30913966 10.1080/19490976.2019.1592421 PMC7524274

[92] Ho GT, Theiss AL. Mitochondria and inflammatory bowel diseases: toward a stratified therapeutic intervention. Annu Rev Physiol. 2022;84:435–59. 34614372 10.1146/annurev-physiol-060821-083306 PMC8992742

[93] Wu S, Feng J, Liu C, Wu H, Qiu Z, Ge J, et al. Machine learning aided construction of the quorum sensing communication network for human gut microbiota. Nat Commun. 2022;13(1):3079. 35654892 10.1038/s41467-022-30741-6 PMC9163137

[94] Wu S, Liu C, Feng J, Yang A, Guo F, Qiao J. QSIdb: quorum sensing interference molecules. Brief Bioinform. 2021;22(4):bbaa218. 33003203 10.1093/bib/bbaa218

[95] Wu S, Yang S, Wang M, Song N, Feng J, Wu H, et al. Quorum sensing-based interactions among drugs, microbes, and diseases. Sci China Life Sci. 2023;66(1):137–51. 35933489 10.1007/s11427-021-2121-0

[96] Oliveira RA, Cabral V, Torcato I, Xavier KB. Deciphering the quorum-sensing lexicon of the gut microbiota. Cell Host Microbe. 2023;31(4):500–12. 37054672 10.1016/j.chom.2023.03.015

